# Supplementary figures and images for: Analysis of a fully infectious bio-orthogonally modified human virus reveals novel features of virus cell entry
Source: PLoS Pathog. 2019 Oct 7;15(10):e1007956. doi: 10.1371/journal.ppat.1007956 (PMC6797222; doi:10.1371/journal.ppat.1007956)

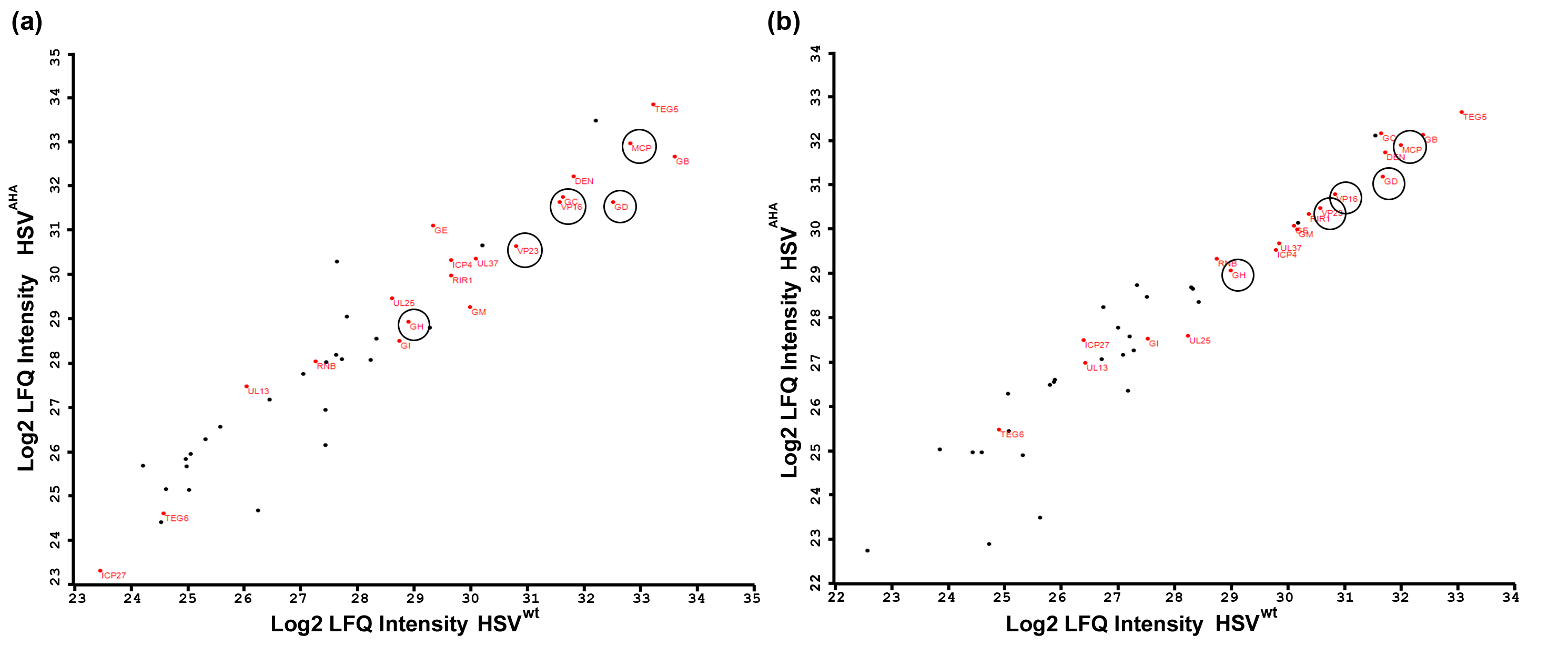

Supplement: S1 Fig — Virus stocks were made during pulse-labelling with the A/M regime (or with Met alone) during single-step (panel a) or multi-step replication (panel b). Equivalent samples of viruses, normalised on the basis of infectivity were lysed, digested with trypsin, and analysed by LC-MS/MS. The results are plotted as the correlation between Log2 LFQ Intensities (approximating protein abundances) measured for proteins of HSVAHA (Y-axis) and HSVwt (x-axis). Proteins for which Met→AHA substitution sites were detected are highlighted in red. Representative proteins discussed in the text are circled. Further Data for Log2 LFQ Intensities are presented in S1 Table. (TIF) [file ppat.1007956.s001.tif]

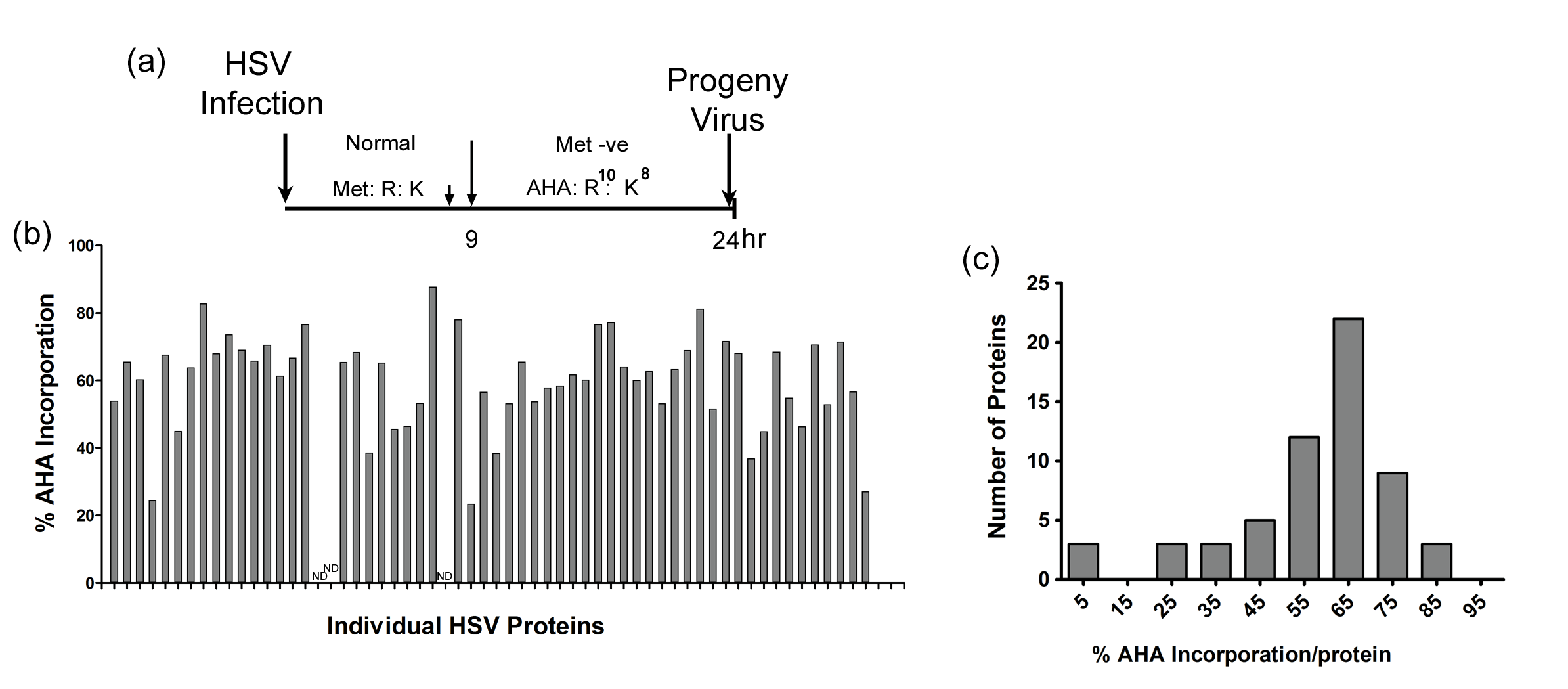

Supplement: S2 Fig — (a) Schematic of labelling regime. Immediately after infection, cells were incubated in standard media. At 8.5 hpi, to deplete pools, this medium was removed and replaced with media lacking Met, Lys, and Arg. At 9 hpi the depletion medium was removed and replaced with media lacking Met, Lys, and Arg but supplemented with AHA, R10 and K8 (the latter two at the normal concentration for DMEM-F12 formulation). Virus was harvested after 24 hpi and virus particles purified and processed for MS. The percentage R10 and K8 incorporation was taken as a surrogate measure for the percentage AHA incorporation during the same labelling interval. (b) The data are illustrated where each vertical bar represents an individual, identified HSV protein and the Y-axis represents the % AHA incorporation into that protein during the labelling interval. ND means not detected. (c) The relative % incorporation for the population of virus proteins was binned into 10% ranges and the number of HSV proteins in each bin then plotted. (TIF) [file ppat.1007956.s002.tif]

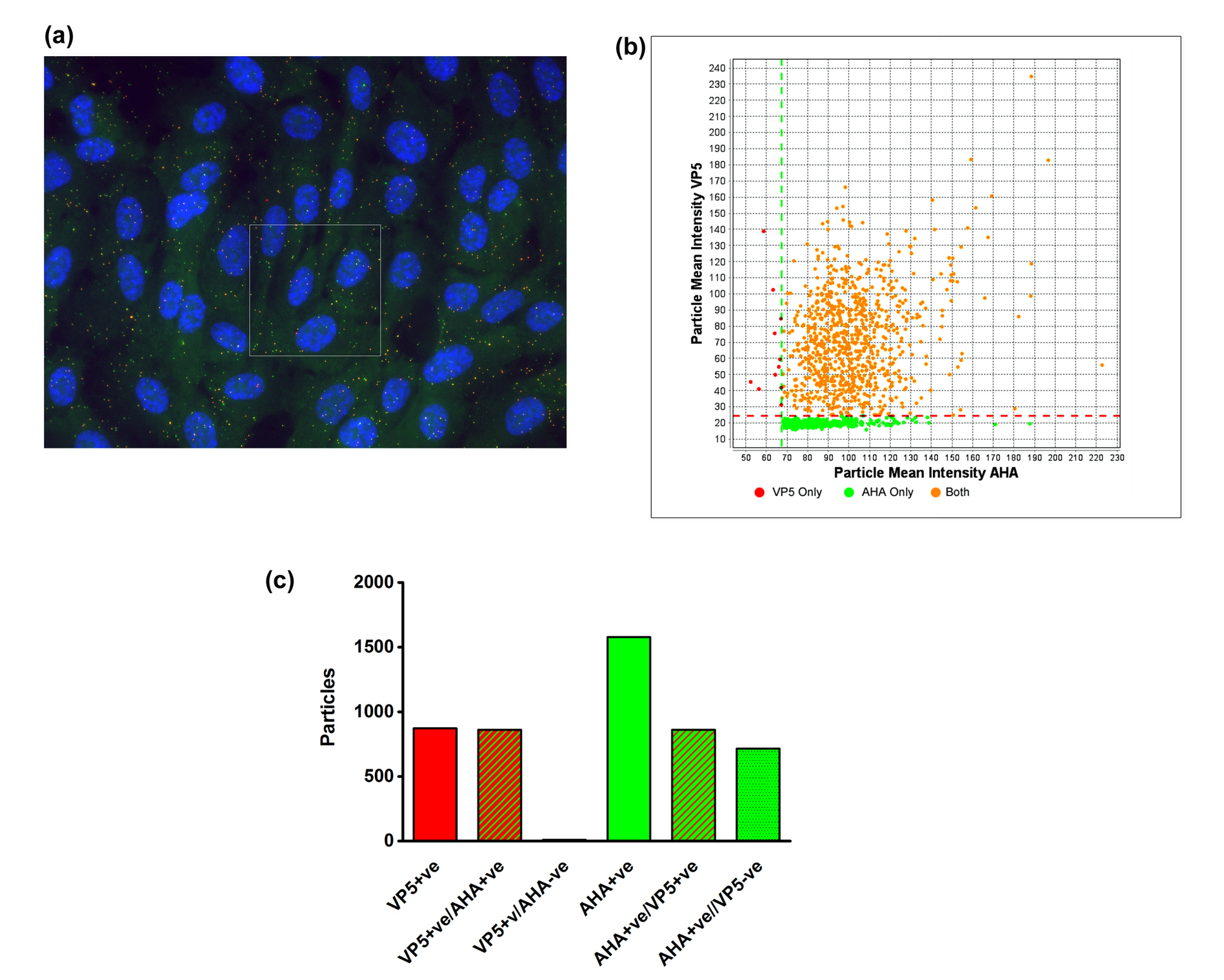

Supplement: S3 Fig — As for Fig 6, cells were infected with HSVAHA and incubated +4°C then fixed immediately. Fig 6A represents the boxed section of the field shown here in panel a. Particles bound to cells at +4°C were detected by CuAAC ligation (green channel) versus detection by anti-VP5 capsids immunofluorescence (red channel). Panel a is a representative field of cells infected at +4°C which was quantitated using Image J as described in methods. Intensities for individual particles (ROIs) in each channel are shown in panel b with Y-axis the VP5 intensity and the X-axis AHA intensity. Each dot in the figure represents a particle ROI which is scored positive in a channel if it is 1 standard deviation above the mean background ROI for that channel (dotted lines). Particles that are positive for both signal are coloured orange, particles that are positive for AHA only are coloured green, and particles that are positive for VP5 only are coloured red. (TIF) [file ppat.1007956.s003.tif]

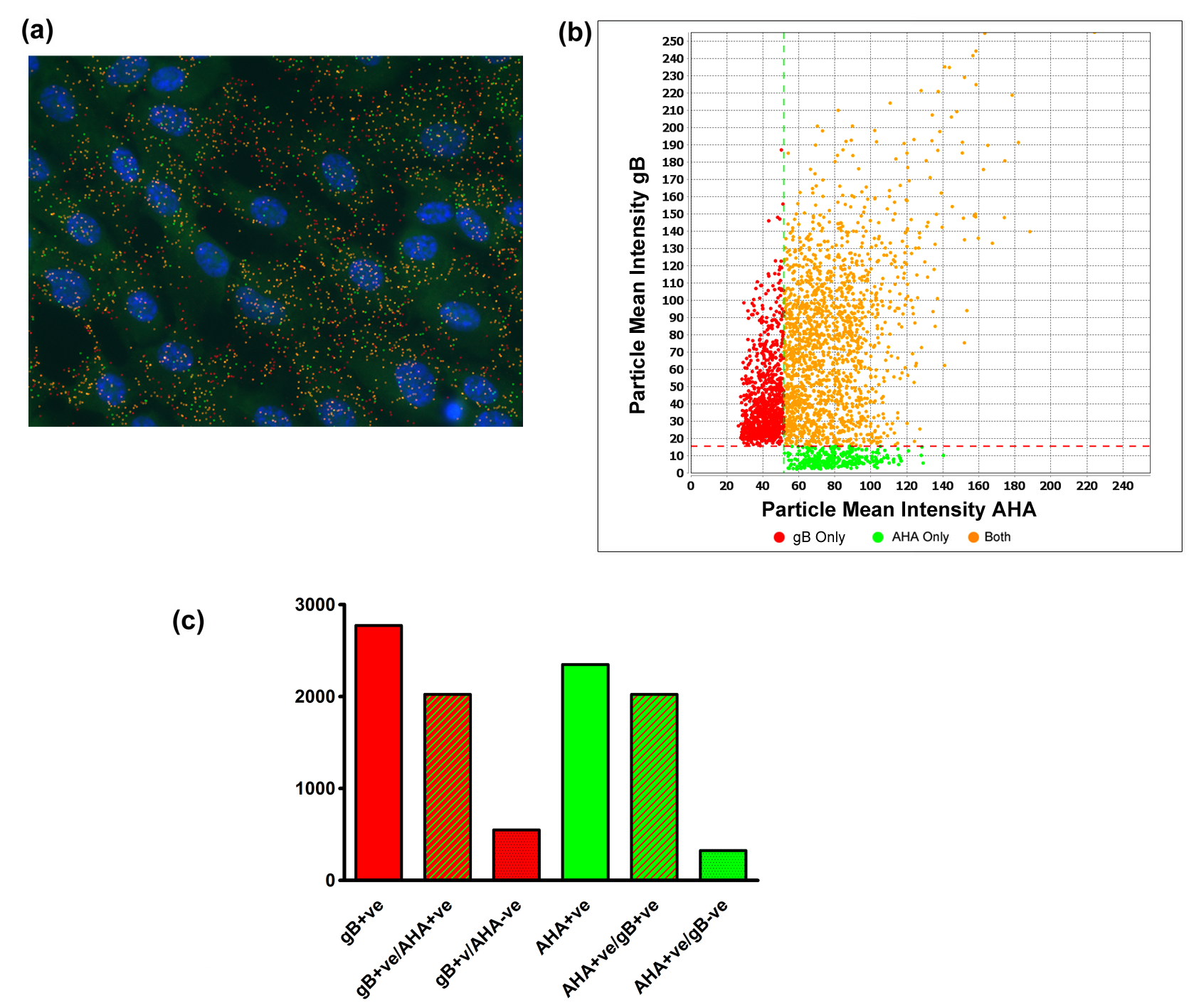

Supplement: S4 Fig — As for S3 Fig, cells were infected with HSVAHA and incubated +4°C then fixed immediately, and processed for detection of AHA signal by click chemistry or gB by immunofluorescence. Panel a shows a field of attached particles scored as described in materials and methods for the presence of both signals (orange), only AHA (green) or only gB (red). Intensities for individual particles are shown in panel b with Y-axis the gB intensity and the X-axis AHA intensity. Numbers of particles above threshold for each category are summarized in panel C. (TIF) [file ppat.1007956.s004.tif]

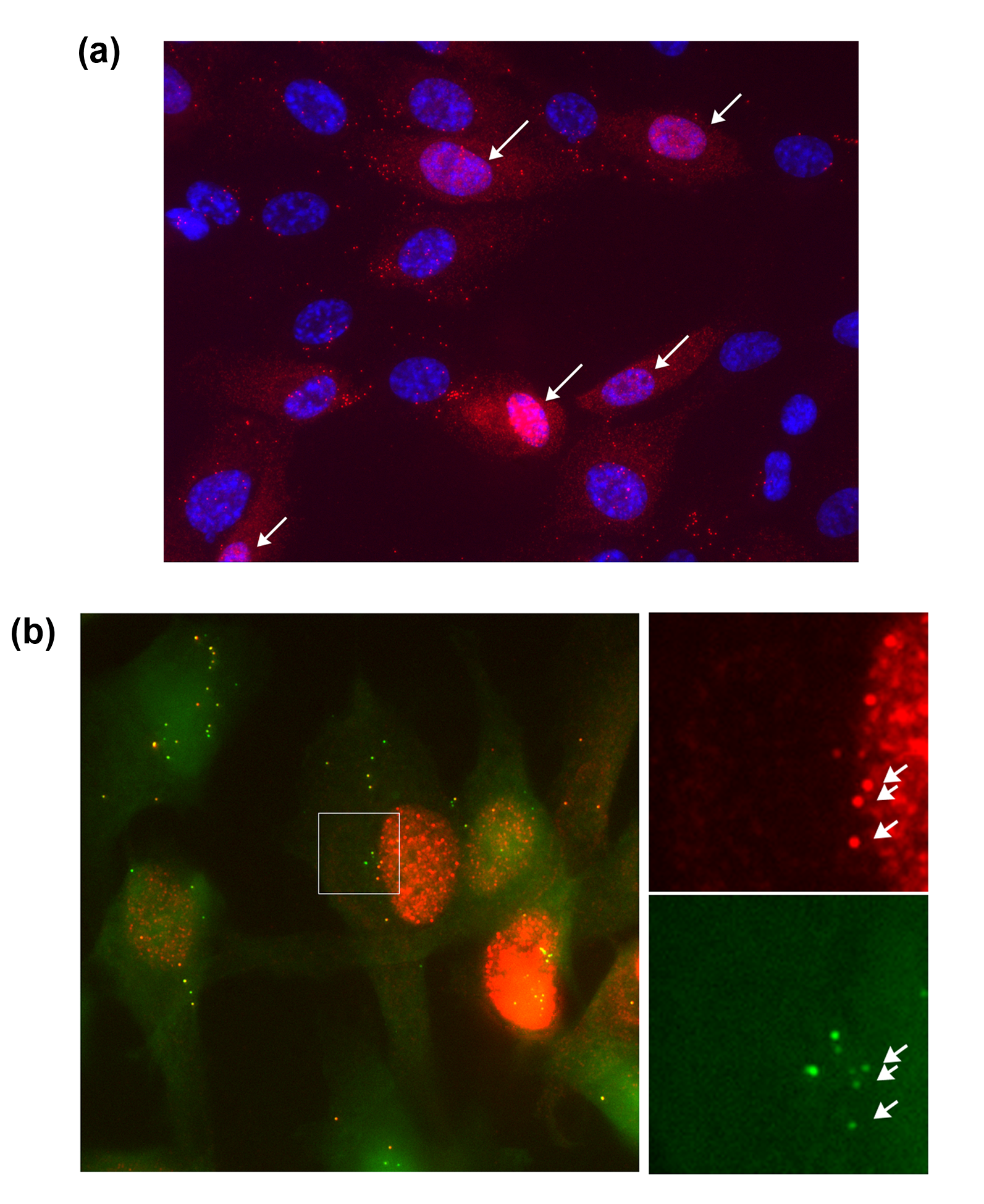

Supplement: S5 Fig — Cells were infected with HSVAHA as normal, shifted to 37°C for 6 hrs, fixed and the distribution of VP5 analysed. Arrows indicate cells with de novo synthesised nuclear VP5 observed at various levels. (b) Cells were infected in the presence of PAA (400 μg/ml) to block virus DNA replication and analysed 6 hpi for VP5 and AHA signals. The boxed area is shown as an inset with cytoplasmic VP5+ve capsids marked by arrows. These capsids are also AHA+ve. (c) An example of cells infrequently observed in the presence of PAA where large numbers of cytoplasmic particles could be observed. The inset shows that in such cases, virtually all capsids were also AHA+ve and thus represented incoming infecting particles. (TIF) [file ppat.1007956.s005.tif]
